# Ant dominance may mask effects of tree diversity on carcass decomposition

**DOI:** 10.1007/s00442-026-06010-4

**Published:** 2026-11-11

**Authors:** Finn Rehling, Matteo Dadda, Marc Nagel, Georg Albert, Helge Bruelheide, Jing-Ting Chen, Heike Feldhaar, Felix Fornoff, Arong Luo, Massimo Martini, Xiao-Yu Shi, Michael Staab, Xianglu Deng, Xiaojuan Liu, Qing-Song Zhou, Chao-Dong Zhu, Alexandra-Maria Klein

**Affiliations:** 1 Chair of Nature Conservation and Landscape Ecology, Albert-Ludwigs- University Freiburg, Stefan-Meier-Str. 76, 79104 Freiburg, Germany; 2 Animal Population Ecology, Bayreuth Center of Ecology and Environmental Research (BayCEER), University of Bayreuth, Bayreuth, Germany; 3 Forest Nature Conservation, Georg-August-University Göttingen, Göttingen, Germany; 4 Institute of Biology/Geobotany and Botanical Garden, Martin Luther University Halle-Wittenberg, Halle (Saale), Germany; 5 German Centre for Integrative Biodiversity Research (iDiv) Halle-Jena- Leipzig, Leipzig, Germany; 6 State Key Laboratory of Animal Biodiversity Conservation and Integrated Pest Management, Institute of Zoology, Chinese Academy of Sciences, Beijing, China; 7 Animal Ecology and Trophic Interactions, Institute of Ecology, Leuphana University Lüneburg, Lüneburg, Germany; 8 Institute of Botany, Chinese Academy of Sciences, Beijing, China

**Keywords:** Biodiversity-ecosystem functioning, Carcass decay, Cadaver, Carrion, Necromass, Scavenger, Postmortem interval, Forensic entomology, Nutrient cycling, Tree species richness, Trophic interactions

## Abstract

Tree diversity promotes the diversity of organisms at higher trophic levels and associated ecosystem functions. Tree diversity may thus also influence communities of insect decomposers and thereby accelerate the decomposition of animal carcasses. To test this, we placed 864 dead mice and measured their decomposition over two days across 96 plots in a forest biodiversity experiment (BEF-China) during the monsoon season in subtropical China in 2024. We also captured carcass-associated arthropods using mouse-baited traps subsequently. We sampled 8,916 decomposer invertebrates (≥ 60 species, twelve orders). The abundance, richness, and composition of decomposers (ants, flies and other) were indirectly linked to tree diversity through associated changes in canopy cover. Ants were most abundant in traps (55.5% of individuals) and dominated carcasses in over half of cases (55.9%), whereas flies, although common (33.1%), rarely dominated decomposition (7.0%). Carcass decomposition was 30.8% faster in closed forests but was not additionally influenced by tree or decomposer species richness. Decomposition was instead strongly related to ant-fly interactions. It was 66.5% faster when flies, rather than ants, were the primary decomposers. When both groups co-occurred, ants often outcompeted flies, resulting in slower decomposition. This study shows that, in subtropical forests during the monsoon season, ants dominate small vertebrate carcasses, suppress other decomposers and respond little to tree diversity. Consequently, decomposition rates are only weakly linked to the richness of decomposer or tree species. These findings highlight that ecosystem functions mediated by higher trophic interactions can depend more on the identity and interactions of dominant species than on overall biodiversity.

**Supplementary Information:**

The online version contains supplementary material available at https://doi.org/10.1007/s00442-026-06010-4.

## Introduction

Tree diversity can enhance ecosystem functioning through mechanisms such as niche partitioning, facilitation, and multitrophic interactions. These mechanisms improve the efficiency of resource use and promote the stability of ecological functions (Trogisch et al. 2021; Liu et al. 2026). Biodiversity-ecosystem functioning (BEF-) experiments have shown that mixtures of tree species increase forest productivity and carbon sequestration (Liu et al. 2018; Huang et al. 2018; Bongers et al. 2021; Depauw et al. 2024). They can also promote the diversity of organisms of higher trophic levels (Fornoff et al. 2019; Schuldt et al. 2019; Chen et al. 2023) and their functions (Vehviläinen and Koricheva 2006; Plath et al. 2012; Fornoff et al. 2019; Beugnon et al. 2023). These insights have made BEF research a foundation for forest restoration and biodiversity conservation (Baeten et al. 2019; Messier et al. 2022). However, BEF experiments have focused predominantly on functions directly linked to plants, leaving ecosystem functions that involve organisms of higher trophic levels understudied (Grossman et al. 2018; Klein et al. 2026). One such example is animal carcass decomposition, a key function for food-web dynamics and energy flow.

**Fig. 1:**
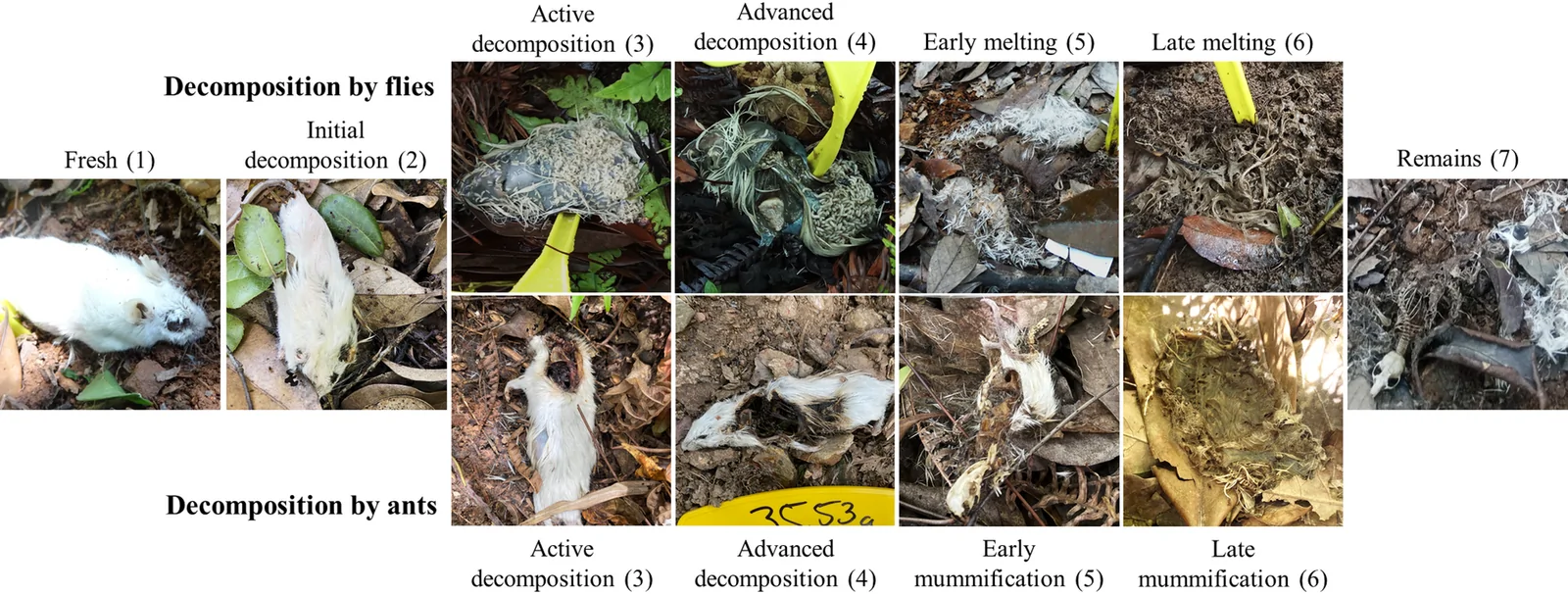
Decomposition pathways of mouse carcasses over time, primarily decomposed by either flies (‘melting’) or ants (‘mummification’) decomposition. Based on the observed decomposition stages in the images, carcasses were assigned a decomposition score ranging from 1 to 7 (see Table S1). When flies decompose carcasses, their larvae secrete digestive enzymes that break down proteins and fats, causing the carcasses to soften and liquefy, effectively ‘melting’ into the ground. In contrast, when ants dominate decomposition, they often cover the carcass with soil or suppress other decomposers, which can lead to mummification. Images by Finn Rehling and Marc Nagel

When an animal dies, its body becomes a concentrated, ephemeral and spatially unpredictable source of energy and nutrients. As such, carcasses are highly attractive to a diverse community of decomposers and scavengers throughout the food web (Carter et al. 2006; Wilson and Wolkovich 2011; Moleón et al. 2026). Arthropods play a central role in carcass decomposition (Payne 1965; Amendt et al. 2004; Paula et al. 2016; Büchner et al. 2024; Wierer et al. 2024), especially in regions where vertebrate scavengers have declined (Oaks et al. 2004; Sonawane et al. 2025). Blow flies (Calliphoridae) and flesh flies (Sarcophagidae) are often among the first to colonize a carcass. They lay eggs or larvae in natural openings, and their larvae rapidly consume the carcass tissue. Their digestive enzymes break down proteins and fats, which softens and liquefies the carcass (Fig. 1). Ants, beetles and a suite of typically phytophagous insects feed on the carcass and prey on fly larvae (Baz et al. 2010; Matuszewski and MĄdra-Bielewicz 2022). Ants can be important scavengers of carcasses in (sub-)tropical forests (Eubanks et al. 2019), where they account for up to 20–60% of arthropod biomass (Schultheiss et al. 2022). Ants oftentimes slow the decomposition of carcasses by suppressing flies, either by disrupting oviposition or by predating eggs or larvae. This interference reduces larval activity, and can lead to carcass drying and eventual mummification (Fig. 1). In some cases, however, ants can also accelerate carcass decomposition by feeding directly on the carcass (Nooten et al. 2022).

Although animal carcasses represent a relatively small portion of the total detritus pool in forest ecosystems (Barton et al. 2019), they are local hotspots of arthropod activity. Carcass decomposition depends strongly on the composition of arthropods present at carcasses and their interactions, but also on the forest environment. As tree diversity positively affects arthropod diversity through increased habitat complexity and resource availability (Schuldt et al. 2019; Wang et al. 2025), it may indirectly affect carcass decomposition through positive effects on the diversity of carcass-associated arthropods (Farwig et al. 2014; Barton and Evans 2017; Marschalek and Deutschman 2022).

However, tree diversity also alters forest structure and microclimate (Schnabel et al. 2025; Deng et al. 2025), which may affect carcass decomposition through pathways other than that of arthropod diversity. For instance, more open forests may increase temperature and arthropod activity and thus accelerate carcass decomposition (Achury et al. 2023). However, carcasses may also dry out, which can limit microbial and arthropod activity and slow decomposition (Finaughty and Morris 2019). In closed forests, cooler conditions may reduce arthropod activity, while higher and less variable moisture conditions may keep carcasses accessible to arthropods and promote decomposition. Thus, the effect of canopy cover on carcass decomposition likely depends on temperature, moisture and arthropod activity, and may thus vary with season (de Carvalho and Linhares 2001; Farwig et al. 2014; Paula et al. 2016; Finaughty and Morris 2019).

The BEF-China experiment (Bruelheide et al. 2014), which experimentally manipulates tree species richness, provides an ideal setting to address these questions. In this study, we investigate how tree species richness affects decomposer communities and small vertebrate carcass decomposition through different pathways. Throughout the manuscript, we define ‘carcass decomposition’ as the combined mass loss caused by arthropod scavenging and necrophagy, and by microbial decomposition. In line with this, we use the term ‘decomposers’ to refer collectively to arthropod scavengers (e.g. ants), necrophagous arthropods (e.g. flies) and microbial decomposers (sensu Moleón et al. 2026). We hypothesized that (i) tree species richness increases the diversity of carcass decomposers (for instance, by providing a wider range of resources and habitats, or by altering forest structure and microclimate) and thereby accelerates the decomposition of animal carcasses; (ii) canopy cover affects decomposer diversity and carcass decomposition. More open forests could either slow decomposition through drying or enhance it by supporting arthropod activity; and (iii) carcass decomposition depends on decomposer diversity, and on the identity of the dominant decomposer group associated with a carcass, especially ants, whose effects may be either negative (through competition with other decomposers) or positive (through direct consumption).

## Materials and methods

### Study area

This study took place in the BEF-China experiment, located in a hilly region near Xingangshan, Jiangxi Province, China (117°54′ E, 29°07′ N). The region has a mean annual temperature and precipitation of 16.7 °C and 1800 mm. The natural vegetation of this region is a subtropical broad-leaved forest with evergreen tree species being predominant. BEF-China encompasses two study sites which were established in 2009 (site A) and 2010 (site B). The two sites are located 4 km apart from each other and together cover 50 ha. This makes BEF-China the currently largest tree diversity experiment in the world (www.treedivnet.ugent.be) (Paquette et al. 2018). The experiment includes 566 study plots of 25.8 × 25.8 m, each planted with 400 seedlings in a 20 × 20 grid with a spacing of 1.29 m between each seedling (0.6 trees m^−^²). Tree species richness varies among plots from 1 to 24 species per plot (1, 2, 4, 8, 16, 24 tree species per plot), drawn from a pool of 40 native broad-leaved species. Each site has a pool of 24 broad-leaved native tree species and eight tree species are shared between sites. All plots were initially weeded twice annually to reduce competing vegetation. The position of trees within plots and the assignment of tree richness levels were randomized. Site A ranges in altitude from 105 m to 275 m a. s. l. and site B from 105 m to 190 m a. s. l. Slopes range from 0° to 45° (site A = 25°, site B = 30°). An extended description of the BEF-China experiment is provided in Bruelheide et al. (2014).

In BEF-China, the number of replicates at each diversity level follows a ‘broken-stick’ design, i.e., each tree species is equally replicated across plots at a given diversity level. Consequently, each site contains more monocultures than forests with higher tree richness. To improve the balance in replication across the tree species richness gradient, we used a subset of 96 plots (48 plots per site) for this study. This subset included 30 monocultures, 16 two-species mixtures, 17 four-species mixtures, 16 eight-species mixtures, 11 sixteen-species mixtures, and all six twenty-four-species mixtures. This subset provided a well-balanced representation of tree species richness levels, and retained monocultures for almost every tree species included in the species-rich mixtures.

### Carcass decomposition experiment

We purchased frozen carcasses of white *Mus musculus* (house mouse) from an online retailer in China. We stored the frozen mice at -18 °C and weighed them before the experiment. The fresh mass of frozen mice was on average 30.6 g, with 95% of individuals ranging from 22.7 g to 44.8 g (Fig. S1). Mouse carcasses represent an ecologically meaningful model of native small rodents (e.g., *Niviventer* spp. and *Apodemus* spp.). Compared with larger carcasses, they also allow for greater experimental replication within and across plots while avoiding excessive nutrient pulses to the experimental setup. We placed three mouse carcasses around each of three randomly selected trees per plot (*n* = 9 carcasses per plot, Fig. S2). The experiment ran for 27 days (between 30 June and 25 July 2024). It was initiated on eleven separate dates, covering an average of nine plots per session (range: 6–17 plots). In total, we deployed 864 carcasses (3 mice × 3 trees × 96 plots across the two sites). The status of ten mice was not investigated. Carcasses were not protected from scavenging by vertebrates (see Anderson et al. 2026). During the experiment, 215 mice (25.2%) were removed and a complete removal of carcasses occurred at six plots.

### Decomposition scores and relative mass loss

We photographed each carcass two days after deployment. Based on the appearance of the carcass in the photos, two observers independently assigned a decomposition score ranging from 1 (fresh) to 7 (only remains; Fig. 1). The scoring system followed established decomposition stages (Payne 1965; Farwig et al. 2014) and was adapted to seven stages. At one plot, photographs were taken after four days instead of two. For these carcasses, the assigned scores were divided by two before the analysis. In addition to assigning scores of decomposition, we recorded the dominant decomposer group. Flies were assigned when carcasses showed signs of liquefication (‘melting’, Fig. 1) or when flies or maggots were visible. Ants were assigned when signs of mummification or ant activity were visible (‘mummification’). When both groups occurred, both decomposers were recorded as dominant. Further details on the characteristics of each decomposition stage are provided in electronic supplementary material (Table S1).

**Fig. 2:**
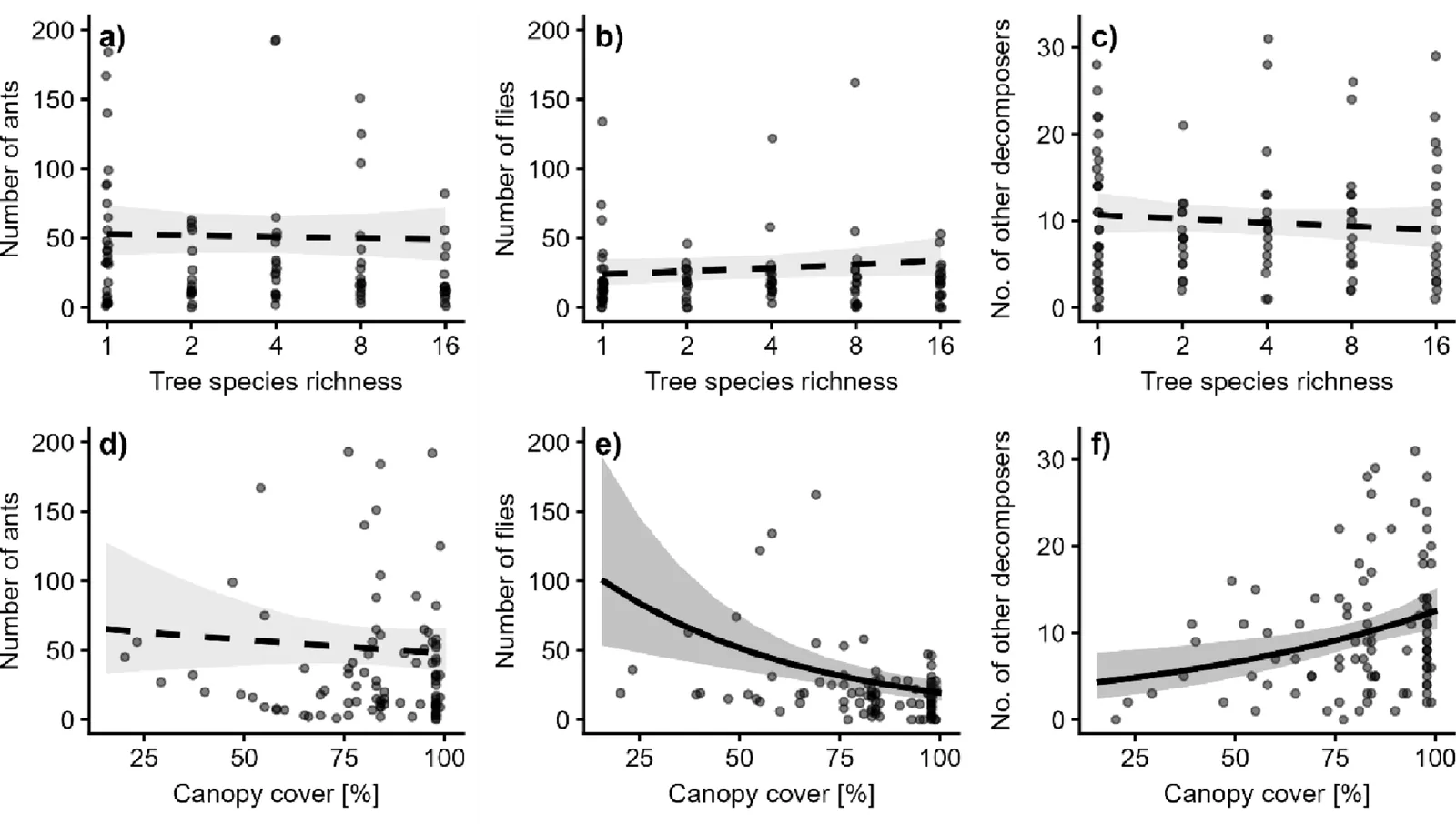
Abundance of ants, flies, and other decomposers in carcass-baited traps in relation to (log2-transformed) tree species richness (**a**, **b**, **c**) and canopy cover (**d**, **e**, **f**). Each point represents one plot. Solid lines indicate statistically significant relationships (*p* < 0.05) with 95% CI as shaded polygons, while grey dashed lines with pale intervals represent non-significant relationships

Before scoring, observers were trained using a set of test photos to harmonize assessment criteria. All photographs were then scored independently. Inter-observer agreement was high across plots and sites (Cronbach’s α = 0.82, *n* = 636). We averaged the decomposition scores across observers.

In a similar experiment conducted in 2023, we freshly weighed a subset of 176 carcasses after two days (site A: *n* = 75, site B: *n* = 101). Relative (fresh) mass loss was strongly correlated with photo-based decomposition scores (GLMM, beta-binomial distribution with log link: R² = 0.965, χ² = 290.7, *P* < 0.001; Fig. 2), validating the scoring approach. We converted the ordinal decomposition scores (x) into a continuous estimate of relative carcass mass using the GLMM parameters (Fig. S3):

1 $$\:\begin{array}{c}relative\:carrion\:mass\:\left(x\right)=\frac{1}{1+{e}^{-\left(3.7183-0.8801*x\right)}}\end{array}$$

### Sampling and identification of arthropod decomposers

One week after completing the decomposition experiment, we deployed one trap baited with a mouse carcass at the center of each plot to sample carcass-associated arthropods (Fig. S4). Each trap consisted of a large plastic container (5 L) and a plastic bottle (0.5 L). This original design is based on a trap used by Farwig et al. (2014) to collect arthropod decomposers. We revised the trap design by suspending the carcass in a plastic cup from a fine metallic wire attached to the top of the large container (Fig. S4). Openings were cut into the lower part of the large container to allow arthropods access to the carcass. The large bottles were perforated at the top to enhance air circulation and the diffusion of volatiles. The smaller container, perforated at the bottom and positioned at a steep angle on top of the large container, was placed on top of the large container to capture flying insects. Traps were partially buried to reduce the probability of being knocked over. The bottles were filled with salt-soap water to reduce surface tension. After two days, we emptied the traps and removed the remaining carcass. We collected all arthropods, rinsed the specimens in 100% ethanol and stored them in fresh 100% ethanol (replaced after 24 and 72 h) at -18 °C until species identification.

All collected specimens were first sorted into morphotypes and counted. Individuals were then identified to the lowest taxonomic level using dichotomous keys (e.g., for ants). When a morphological identification was not feasible, DNA barcoding was applied. For each morphotype selected for barcoding, one leg from two representative individuals was sampled. DNA was extracted and a fragment of the mitochondrial Cytochrome c oxidase subunit I (COI) gene was amplified using the primers LCO1490 and HCO2198. PCR products were sequenced following standard Sanger protocols (Sanger et al. 1977; Hebert et al. 2003). Sequences were compared with records in the NCBI nucleotide database (https://www.ncbi.nlm.nih.gov/).

We quantified sample coverage of the carcass-baited traps following Chao et al. (2020). Traps captured nearly complete necrophagous communities of flies and ants (Fig. S5), whereas other decomposer groups were underrepresented. Sample coverage was 90.0% for flies, 90.4% for ants, and 60.7% for other decomposers. Coverage estimates were only calculated using traps that collected at least five individuals of at least two species within a decomposer group (see Statistical analysis).

### Canopy cover

Canopy cover for each plot was estimated using elevation-normalized LiDAR point clouds collected in 2023. Additional drone-based LiDAR data were acquired during the leaf-on season in September 2024 using a Hesai Pandar40P laser scanner mounted on a LiAir 220 N system (GreenValley International). The drone flew at an average altitude of 100 m and a velocity of 8 m/s, yielding an average point density > 164.6 points m^− 2^. Most flight lines overlapped > 50%, and the maximum scan angle was ± 15°. LiDAR data were pre-processed using LiDAR360 software (GreenValley International, Beijing, China) following a standardized workflow (Liu et al. 2024). Canopy cover was calculated as the proportion of ground (above 2 m) covered by forest canopy, approximately the inverse of the gap fraction.

### Statistical analysis

#### Decomposer abundance

Because the contribution of arthropods to carcass decomposition varies among groups, we analyzed necrophagous flies, ants and other decomposers separately. To avoid bias from undersampling, we a priori excluded traps from analyses of a given group if they contained no species, only one species, or fewer than five individuals (i.e., 16 traps for flies, 12 traps for ants, and 30 traps for other decomposers). Traps could still be used for analyses of other groups. We analyzed the relationships between the abundance of arthropods and canopy cover and log2-transformed tree species richness with (generalized) linear mixed effect models (type III sums of squares). We used a negative binomial error distribution with a log link to account for overdispersion in count data. We calculated values of estimated species richness with iNEXT (Hsieh et al. 2016) for standardized sample coverage of 90% for flies and ants, and a coverage of 60% for other decomposers. In these models, we used a gamma error distribution with log link. We included the experimental site (i.e., A or B) and geomorphological type of the plot (e.g., slope, ridge, valley) as a random intercept. We z-transformed all continuous fixed factors (to mean = 0 and SD = 1). We checked multicollinearity visually and using variance inflation factors before analyses.

The relationship between the species composition of different groups of arthropods (flies, ants, and other decomposers) in carcass-baited traps (response variable) and log2-transformed tree species richness, canopy cover, and geomorphological position of a plot (fixed factors) was analyzed using permutational analysis of variance (PERMANOVA) (Anderson 2017). All continuous fixed factors were z-transformed before analysis. The abundance of species was Hellinger-transformed before analysis. The Bray-Curtis index was calculated as a measure of dissimilarity between samples. To account for the nested structure of the design, arthropod samples were permuted within study sites, with 9999 permutations used to calculate a quasi-F statistic.

#### Carcass decomposition analysis

After converting the decomposition scores into values of relative mass loss using Eq. (1), we used a generalized linear mixed model to investigate the effects of log_2_-transformed tree species richness, canopy cover and the identity of primary decomposers (based on photos) on the proportional mass loss of carcasses after two days. We used a beta family error distribution with a logit link to account for non-normality and heteroscedasticity. We included plots, the tree species around which the carcass was placed, and geomorphological type of the plot (i.e., slope, ridge, valley) as a random intercept. Initial mass of mouse carcasses was not related to their decomposition and did not alter the influence of other variables, so it was excluded. Dispersion was modelled as a function of canopy cover and primary decomposer to address heterogeneity in variability. All continuous predictors were z-transformed (to mean = 0 and SD = 1) before analysis. To investigate the relationship between ant dominance in carcass decomposition and ant abundance, we fitted a linear mixed-effects model with a binomial error distribution and a logit link function. Ant presence on carcasses was included as the response variable, (log-transformed) ant abundance as a fixed effect, and site as a random effect.

#### Structural equation model

To investigate the relationships between tree species richness, canopy cover, decomposer abundance and carcass decomposition jointly, we fitted a piecewise structural equation model. Because decomposer abundance was measured at the plot level, we aggregated carcass observations within plots to relate ant and fly abundance to their role as primary decomposers and to carcass decomposition. We calculated the mean relative carcass mass loss and the proportion of carcasses dominated by ants for each plot. For flies, we used a binary response indicating whether flies were recorded as primary decomposers on at least one carcass within a plot.

The component models included the predictors used in the preceding analyses, with additional paths linking decomposer abundances (from the baited traps), primary decomposers (based on the photos) and carcass mass loss (see Fig. 4, Table S7). We included study site as a random intercept in all component models. We included a correlated error between log_10_-transformed ant and log_10_-transformed fly abundance without specifying a causal direction as we had insufficient information on potential ant–fly interactions within, at the entrance to, or outside the traps. The proportion of carcasses primarily decomposed by ants was included as a predictor of fly occurrence, to investigate whether flies decomposed primarily carcasses less frequently in plots with greater ant dominance. We used Gaussian error distributions with identity links for canopy cover and decomposer abundances. Ant dominance was analyzed using a binomial error distribution with a logit link, weighted by the number of assessed carcasses per plot. For fly occurrence as primary decomposers, we used a binomial error distribution with a logit link. Mean relative carcass mass loss was analyzed using a beta error distribution with a logit link. Overall model fit was assessed using Fisher’s C and the generalized chi-squared goodness-of-fit test.

All statistical analyses were done with R program version 4.4.1 (Team 2021). Generalized linear mixed models were constructed using the R-package ‘glmmTMB’ version 1.1.10 (Brooks et al. 2017). Multicollinearity among predictor variables was assessed using ‘performance’ version 0.12.4 (Lüdecke et al. 2021). Significance values were obtained using Wald-χ2-tests in the package ‘car’ version 3.1.2 (Fox and Weisberg 2019). Model performance was evaluated using the R-package ‘DHARMa’ version 0.4.7 (Hartig 2024). Species richness estimation and sample coverage calculations were performed using the R-package ‘iNEXT’ version 3.0.1 (Hsieh et al. 2016). The multivariate analysis was conducted using the R-packages ‘vegan’ version 2.6.6.1 (Oksanen et al. 2020), with ‘RVAideMemoire’ version 0.9.83.7 (Hervé 2022) used for type II PERMANOVA tests and permutation. Models with tree species richness as the only predictor are presented in the Supplementary, as these directly reflect the experimental treatment of the sites (Supplementary Text S1, Tables S3–S4). The piecewise SEM was conducted with the R-package ‘piecewiseSEM’ (Lefcheck 2016).

## Results

### Decomposer diversity

In 2024, we collected 4,946 ants representing 13 species, 11 genera and four subfamilies in BEF-China. The five most abundant species were *Crematogaster cf. rogenhoferi* (26.2%), *Pheidole nodus* (17.3%), *Polyrhachis illaudata* (sensu lato, 14.7%), *Polyrhachis dives* (10.2%), and *Lepisiota* sp. (7.6%). All other carcass-associated ants each accounted for less than 7% of individuals (see Supplementary Table S2). The abundance of ants in carcass-baited traps did not change with tree species richness (Wald-χ² = 0.080, *P* = 0.778) or canopy cover (Wald-χ² = 0.55, *P* = 0.458, Fig. 2a/d). The species richness of ants only increased with canopy cover (Wald-χ² = 4.40, *P* = 0.036), but not with the number of ants (Wald-χ² = 0.63, *P* = 0.428) nor tree species richness (Wald-χ² = 1.14, *P* = 0.287, Fig. S6 d-f). The community composition of ants was not related to the forest environment (Table S5).

We further collected 2,953 flies representing 12 species, nine genera, and five families. The three most abundant species were *Sarcophaga peregrina* (34.7%), *Hemipyrellia ligurriens* (24.6%) and *Chrysomya megacephala* (22.9%). Calliphoridae were the most frequent family (*n* = 1,739), followed by Sarcophagidae (*S. peregrina* only; *n* = 1,024) or Muscidae (*n* = 185). Fly abundance was not affected by tree species richness (Wald-χ² = 1.63, *P* = 0.201), but decreased with increasing canopy cover (Wald-χ² = 16.32, *P* < 0.001, Fig. 2b/e). Species richness of flies was positively associated with their abundance at the plot level (Wald-χ² = 12.58, *P* < 0.001, Fig. S6 a-c). The community composition of flies showed a marginal relationship with canopy cover (r² = 6.5, F = 5.38, *P* = 0.053; Table S5).

In addition to flies and ants, we collected 1,017 other invertebrates representing at least 47 taxa across 12 insect orders, six spider families and one centipede family. Most individuals belonged to Coleoptera (64.3%), Lepidoptera (14.1%), and Orthoptera (12.2%). The most abundant beetles were *Onthophagus* sp. (35.8%), *Aleochara postica* (10.9%), and *Copris* sp. (9.1%). Among Lepidoptera, the most common were an unidentified morphospecies (4.5%), *Glyphodes* sp. (3.3%), and *Sypnoides fumosa* (2.1%). Orthopterans included *Xenogryllus marmoratus* (5.6%) and *Diestramima excavata* (3.1%), with cockroaches, hemipterans, dermapterans and scutigerid centipedes at low abundances (Supplementary Table S2). The abundance of other decomposers was positively associated with canopy cover (Wald-χ² = 10.81, *P* = 0.001), and was not related to tree species richness (Wald-χ² = 0.51, *P* = 0.474, Fig. 2c/f). A more detailed group-specific analyses indicated that the positive association between the abundance of other decomposers and canopy cover was primarily driven by positive responses of Coleoptera and Lepidoptera (Table S6). Species richness of other decomposers was strongly associated with their abundance at the plot level (Wald-χ² = 6.74, *P* = 0.009), but did not depend on the forest environment (Fig. S6 g-i). The community composition of other decomposers showed a marginal relationship with canopy cover (r² = 6.8, F = 5.20, *P* = 0.027, Table S5).

### Carcass decomposition

**Fig. 3:**
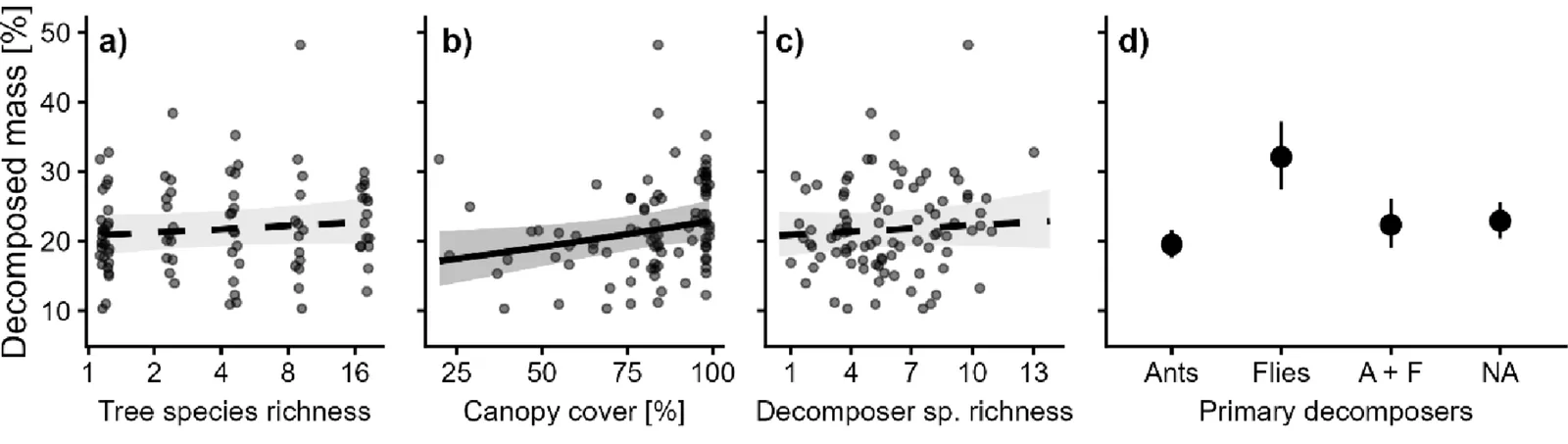
The lost mass of decomposing carcasses after two days in the subtropical forest of BEF-China in relation to (**a**) tree species richness, (**b**) canopy cover, (**c**) decomposer species richness and (**d**) the primary decomposer (ants, flies, ‘A + F’ = ants and flies, ‘NA’ = unknown). Each point represents one plot. Solid lines indicate statistically significant relationships (*p* < 0.05) with 95% CI as shaded polygons, while grey dashed lines with pale intervals represent non-significant relationships. In (d), the error bars indicate 95% CI

In BEF-China, the carcasses were on average 22.2% decomposed after two days, with 95% of values ranging from 7.0% to 45.6% (Fig. 3). In a model including only tree species richness, relative mass loss was positively influenced by tree species richness (Wald-χ² = 4.01, *P* = 0.045). However, this effect was no longer detectable when canopy cover and decomposer-related variables were included in the model (Wald-χ² = 1.25, *P* = 0.264), while canopy cover had a positive effect (Wald-χ² = 5.99, *P* = 0.014). Consistently, carcass decomposition increased from 17.4% in open forests to 22.8% in closed forests. Decomposer species richness had no detectable effect (Wald-χ² = 0.82, *P* = 0.365), whereas decomposition was strongly dependent on the identity of the primary decomposer (Wald-χ² = 57.38, *P* < 0.001).

Ants completely buried carcasses with soil in 47 of 639 cases (7.4%), effectively excluding flies in 42 of these instances (89.4%). Carcasses primarily decomposed by ants decomposed more slowly than those decomposed by flies (Fig. 3d). When ants and flies co-occurred on carcass, the decomposition rates were similar to those of carcass dominated by ants. The probability that a carcass was dominated by ants increased with the number of ants present in the plot (Wald-χ² = 5.95, *P* = 0.015).

### Decomposition pathways

**Fig. 4:**
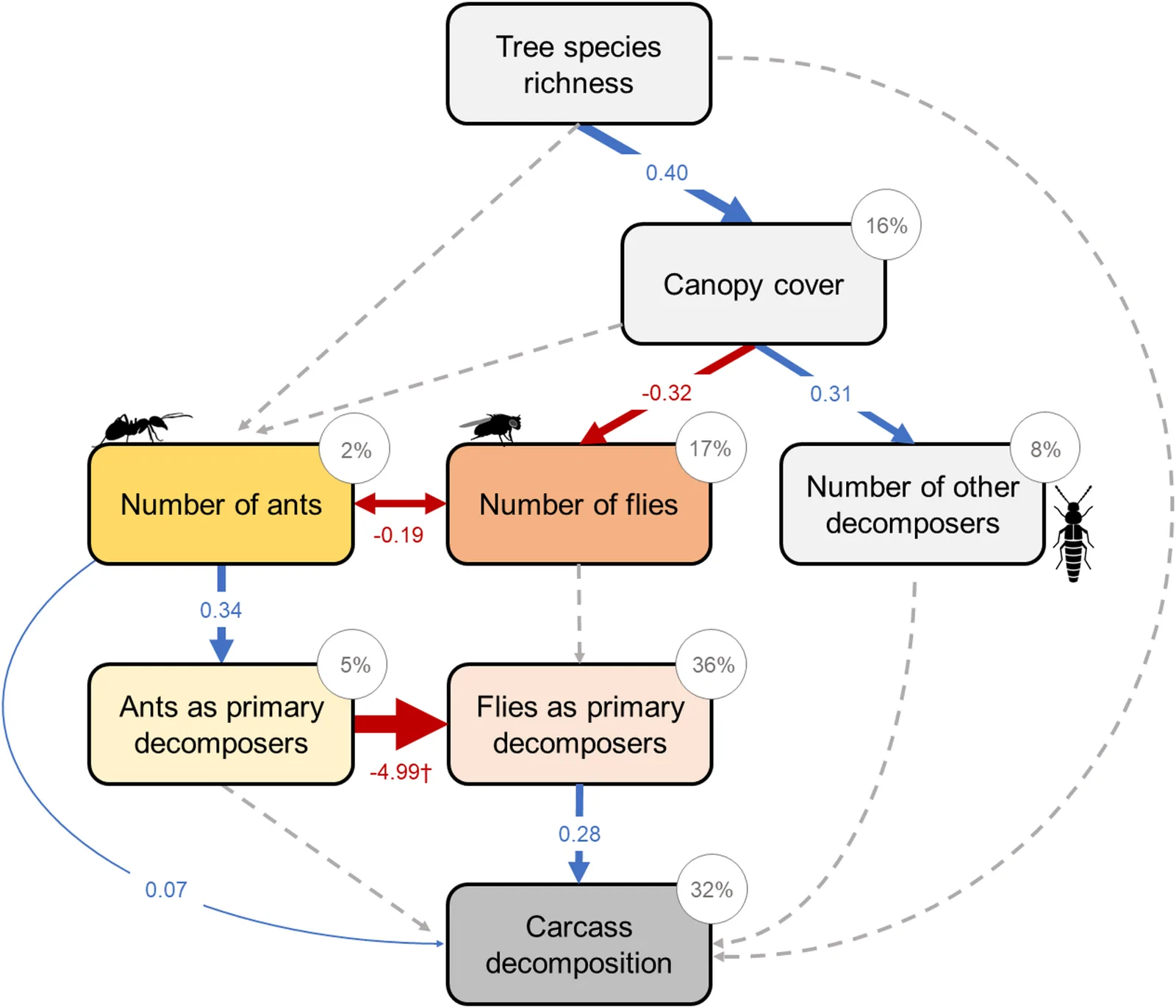
Piecewise structural equation model linking tree species richness, canopy cover, decomposer abundances and carcass decomposition across 90 plots and two sites in BEF-China. Blue and red arrows indicate significant positive and negative relationships, respectively (*P* < 0.05). Grey dashed arrows show selected non-significant paths. Other non-significant paths are omitted for clarity. Labels show unstandardized coefficients on the response scale for Gaussian models and the logit scale for binomial (†) and beta models. The coefficients are not directly comparable across models. The double-headed arrow represents a residual correlation. Percentages indicate marginal R². No lack of fit was detected (Fisher’s C = 4.207, df = 4, *P* = 0.379). For more information, see Table S7

The relationships in the piecewise structural equation model were largely consistent with the preceding mixed-effects analyses (Fig. 4, Table S7). Overall, the model showed no evidence of lack of fit (Fisher’s C = 4.207, df = 4, *P* = 0.379; χ² = 2.191, df = 2, *P* = 0.334). Tree species richness was positively associated with canopy cover (*P* < 0.001), but showed no additional direct relationships with decomposer abundances or carcass mass loss. Unlike the carcass-level mixed model, the structural equation model detected no direct relationship between canopy cover and carcass mass loss after accounting for the abundance of decomposers in traps and the role of primary decomposers at carcasses. Ant and fly abundances in carcass-baited traps showed a negative residual association (*r* = − 0.19, *P* = 0.037). Fly occurrence as primary decomposers decreased with ants as primary decomposers (*P* < 0.001) and was positively associated with carcass mass loss (*P* < 0.001). After accounting for primary decomposers, carcass mass loss was positively associated with ant abundance in traps (*P* = 0.039).

## Discussion

Animal carcass decomposition has received comparatively little attention in experimental biodiversity–ecosystem functioning (BEF) research, which has predominantly focused on plant-related functions and substrates such as leaf litter (Scherer-Lorenzen 2008; Seidelmann et al. 2016; Desie et al. 2023). To our knowledge, our study is the first to investigate the effects of tree species richness on carcass decomposition. Carcass decomposition was mainly influenced by canopy cover and ant–fly interactions. Tree species richness was positively associated with canopy cover, but showed no additional relationship with carcass decomposition. Ants strongly suppressed fly colonization, and perhaps also other decomposers. Our findings suggest that these interactions slowed carcass decomposition and may limit how strongly forest conditions and decomposer diversity affect decomposition. Through such antagonistic interactions, ants may therefore weaken positive biodiversity–ecosystem functioning relationships in subtropical forests.

### Lack of independent effect of tree species richness on decomposer and decomposition

Tree species richness showed a positive effect on carrion decomposition in models including only tree species richness as a predictor. However, when accounting for forest structure (here, canopy cover), we found no independent effect of tree species richness on the decomposer community nor on carcass decomposition. This result contrasts with many BEF-studies, which commonly report positive effects of tree diversity on the diversity of higher trophic levels and their associated ecosystem functions. For instance, tree diversity has been shown to enhance arthropod diversity by increasing habitat complexity and resource heterogeneity (Schuldt et al. 2019; Wang et al. 2025). However, recent work highlights that BEF relationships can be context-dependent: diversity effects differ between ecosystem functions (Tang et al. 2022), can vary over space and time (Trogisch et al. 2021; Bongers et al. 2021; Amyntas et al. 2023), and may be overridden by abiotic conditions or biotic interactions (Li et al. 2023). As a result, BEF-relationships may weaken or vanish at certain ecological scales, which challenges the application of BEF-principles in real-world ecosystems (Manning et al. 2019).

In this study, the two main decomposer groups (ants and flies) showed little response to tree diversity independent of canopy cover. Ants, the most important group of decomposers, are mainly ground-active and omnivorous. Many of these species have broad diets and flexible nesting behaviour (Blüthgen and Feldhaar 2009). Their resources may therefore be less closely linked to tree species identity than those of more specialized consumers (Staab et al. 2014; Skarbek et al. 2020; Spitz et al. 2026). These dominant generalists occur throughout the gradient of tree species richness and may control access to carcasses, leaving little opportunity for tree species richness to influence carcass decomposition.

Although BEF-China belongs to the oldest forest BEF-experiments in the world (Liu et al. 2026), the forest is still relatively young (~ 15 years). Ant communities may therefore still reflect topography and early site conditions (Spitz et al. 2026). The young age of the forest may thus mask diversity effects through spatial legacies (Cole et al. 2016; Hoenle et al. 2022). However, studies from other tropical forests suggest that ant communities recover quite rapidly during forest succession (Grella et al. 2026; Metz et al. 2026).

Flies also did not respond to tree diversity. Mobility and habitat use may explain the weak response. Many carcass flies travel over 2 km per day, especially species that use carcasses for oviposition (Braack and Retief 1986; Spradbery et al. 1995; Smith and Wall 1998). Carcasses may therefore act as local activity hotspots, attracting insects from beyond the immediate tree neighborhood. However, mobility does not preclude responses to local conditions: other mobile taxa respond positively to tree diversity in the context of BEF experiments (Milligan and Koricheva 2013; Fornoff et al. 2019; Xie et al. 2026; Anderson et al. 2026), and fly diversity and composition were related to canopy cover in our study. Future studies should therefore examine whether carcass-associated flies and other decomposers respond more strongly to tree species richness at larger spatial scales.

Tree diversity also showed no independent effect on other decomposers. Flies or ants may have further outcompeted other decomposers or suppressed their activity (Trumbo and Bloch 2000), and potentially limited their contribution to carcass decomposition. These findings suggest that tree species richness plays a limited role in shaping decomposer communities and carcass decomposition when ants​ dominate the process.

### Competition between flies and ants for carcass

It is well established that flies and ants are among the most dominant invertebrate decomposers of carcass and that they often compete directly for access to this ephemeral resource (reviewed by Eubanks et al. 2019). In our study, we observed strong competition between ants and flies for carcasses. During the early monsoon season of 2024, ants usually dominated the carcass. This period was characterized by high rainfall and high humidity, which likely suppressed fly activity and abundance (Arnaldos et al. 2001; de Carvalho and Linhares 2001; Shi et al. 2009). In contrast, ant abundance often increases during the wet season in (sub-)tropical forests (Levings 1983; Suwabe et al. 2009; Queiroz et al. 2023; Kass et al. 2023). In our study, the seasonal conditions likely promoted the role of ants in carcass decomposition and influenced the decomposition rates, respectively.

Ants used a range of physical and behavioral strategies to interfere with flies: in our study, ants buried carcasses with soil in about 8.2% of all cases (i.e. 13.1% of cases dominated by ants). Burial effectively excluded flies in 89% of these instances (cf. Cornaby 1974; Lindgren et al. 2011). When carcasses remained uncovered, ants attacked adult flies, likely reducing oviposition and larval development (Stoker et al. 1995). Ants also preyed on fly larvae directly (Early and Goff 1986; Stoker et al. 1995; de Jong and Hoback 2006; Barton and Evans 2017). These different behaviors likely influence the decomposition process, and depend on ant species.

Overall, carcasses dominated by ants decomposed more slowly than those dominated by flies. This supports previous findings that ants slow decomposition by suppressing flies (Barton and Evans 2017; Pereira et al. 2017; Lin et al. 2022; Wei et al. 2023). Our results further link this pattern to ant abundance: ant dominance increased with ant abundance within plots and was negatively associated with flies occurring as primary decomposers. Early carcass discovery and rapid recruitment of nestmates may allow ants to monopolize carcasses before flies have established (Lindgren et al. 2011). Fly abundance itself did not predict whether flies acted as primary decomposers, suggesting that ants restricted their contribution to decomposition. Nevertheless, ant abundance was weakly positively associated with carcass decomposition after accounting for ant–fly relationships, which may reflect direct consumption by ants (Nooten et al. 2022). Future studies on the differences in ecological strategies among ant species may help explain when ants accelerate carcass decomposition and when their negative effects on other decomposers prevail (Eubanks et al. 2019).

### The role of canopy cover

Open forests are typically warmer and drier, potentially promoting insect activity and diversity (Perry et al. 2018; Wu et al. 2021; Achury et al. 2023; Rothacher et al. 2025). In case of carcass decomposition, open forests may also facilitate carcass detection by decomposers through improved volatile emissions (Von Hoermann et al. 2022). In our subtropical study system, however, warmer and drier conditions in more open forests accelerated carcass drying, which reduced its suitability for colonization by decomposers (cf. Staab et al. 2022; Rehling & Senger et al. 2026). In contrast, cooler and moister conditions in closed forests likely favored colonization and larval development. This potentially resulted in the consistently faster decomposition of carcass in closed forests in our experiment.

Beyond, canopy cover was also related to the community composition of flies and other decomposers. While Barton and Evans (2017) observed no canopy effect on fly community composition, we found evidence of compositional shifts of decomposers, suggesting environmental filtering may vary in strength across seasons or years and systems (Farwig et al. 2014; Büchner et al. 2024; Wierer et al. 2024). Overall, our results align with previous evidence that decomposition proceeds more rapidly in closed compared to open forests (de Carvalho and Linhares 2001; Alvarado-Montero et al. 2021). However, canopy cover showed no additional relationship with decomposition after accounting for decomposer abundances and their roles at carcasses in the plot-level path model. These decomposer variables may therefore account for some of the variation previously attributed to canopy cover, although data aggregation to the plot level may also contribute to this difference. Nonetheless, few studies have directly linked decomposer diversity, forest structure, and decomposition outcomes. Together, these findings support previous evidence that decomposer interactions can be stronger drivers of carcass decomposition than habitat conditions (Barton and Evans 2017), while forest structure may partly influence these interactions through its effects on microclimate and decomposer communities.

## Conclusion

Our study provides new insights into carcass ecology in subtropical forests. During the monsoon season, ants dominated carcasses, and their antagonistic interactions with other decomposers (especially flies) were more closely associated with carcass decomposition than canopy cover or tree and decomposer species richness. These results support the notion that biodiversity–ecosystem functioning relationships may weaken at higher trophic levels because interactions among dominant consumers outweigh diversity effects. Future experiments that manipulate access to carca​sses​ by key taxa can help clarify the relative importance of bottom-up and top-down processes in carcass decomposition.

## Supplementary Information

Below is the link to the electronic supplementary material.

Supplementary Material 1

## Data Availability

The dataset is openly available at Figshare (https://doi.org/10.6084/m9.figshare.30490391) and via the BEF-China data portal (https://data.botanik.uni-halle.de/bef-china). Data can also be accessed via GitHub (https://github.com/nature-rehling/).

## Code availability

Code can be openly accessed via GitHub (https://github.com/nature-rehling/).
