# Supplementary material for: Ant dominance may mask effects of tree diversity on carcass decomposition
Source: Oecologia. 2026 Nov 11;208(12):168. doi: 10.1007/s00442-026-06010-4 (PMC13751805; doi:10.1007/s00442-026-06010-4)
Supplement: Supplementary file 1 [file 442_2026_6010_MOESM1_ESM.docx]

**Supplementary Material: Ant dominance may mask effects of tree diversity on carcass decomposition**

**Author(s): Finn Rehling, Matteo Dadda, Marc Nagel, Georg Albert, Helge Bruelheide, Jing-Ting Chen, Heike Feldhaar, Felix Fornoff, Arong Luo, Massimo Martini, Xiao-Yu Shi, Michael Staab, Xianglu Deng, Xiaojuan Liu, Qing-Song Zhou, Chao-Dong Zhu, Alexandra-Maria Klein.**

**Figure S1**: Distribution of the mass of mouse carrion used in the carrion experiment before the experiment began in 2024.

**Figure S2**: The frequency of locations (i.e. where mice carcasses were placed around trees) at the 96 plots of BEF-China. Each plot consists of a grid of 20 × 20 planted trees, and three mice were laid out around each of three trees. The grey shaded area represents the intensively investigated center of the plots, which was largely avoided in order to prevent olfactory conflicts between multiple, co-occurring experiments.

Table S1: Decomposition scoring scheme for mouse carcass decomposition: A seven-stage classification, depending on whether the mouse carcass was primarily decomposed by flies or ants.

Fresh – Score 1

- Eyes decomposed
- Fur intact

Initial decomposition – Score 2:

- Fur starts to get loose
- Body often bloated
- With few, small (< = 2) holes

Active decomposition – Score 3:

Decomposition by flies:

- Fur mostly loose
- Meat is largely remaining
- Flesh is colored light grey
- Many small maggots visible

Decomposition by ants:

- Dry (but if it was rainy)
- Opened up clean
- Tissue with many (> 2) holes
- Sometimes fur intact, but mostly loose
- Rarely skeleton visible
- If large holes exist, they occur only on

one part of the body

Advanced decomposition – Score 4:

Decomposition by flies:

- Intermediate-sized maggots
- Tissue largely gone
- Mouse-like structure often gone
- Meat often dark grey

Decomposition by ants:

- Skeleton is partially visible
- Meat often gone, or black
- Tissue with large hole (1/4 body)

**Early melting – Score 5 –**

Decomposition by flies:

- Carrion melts into the ground
- Large maggots
- Tissue is still remaining and wet (!)
- Skeleton often detached from tissue
- Often spread in larger area

**Early mummification – Score 5:**

Decomposition by ants:

- Skeleton / spine visible without flesh
- Meat largely gone
- Often some fur remains

**Advanced melting – Score 6:**

Decomposition by flies:

- Tissue largely gone
- Parts of soil moist (!) because maggots

  went into ground
- Almost no signs of maggots

**Advanced mummification – Score 6:**

Decomposition by ants:

- Mummified, dry
- Low activity, few organisms
- Some few

**Remains – Score 7:**

Decomposition by flies:

- Everything is gone
- No activity of any organism
- Skeleton, fur and small parts of tissue remain
- Soil around the carrion is dry

**Figure S4**: Photograph of a baited trap used to sample carcass-associated arthropods in 2024. The trap consisted of a large plastic container (5 L) and a small plastic bottle (0.5 L). A dead mouse was placed in a plastic cup within the trap. Small openings at ground level allowed arthropods to access the carrion, while the small bottle at the top of the large bottle collected flying insects. Both bottles were filled with salt-soap water to preserve the arthropods, and traps were emptied after two days.

**Figure 3**: Remaining mass of carcass [%] in relation to the assigned decomposition score based on the appearance of the mouse carcass in a photograph taken after two days of decomposition in BEF-China. Predicted mean ± 95% CI.

**Figure S5**: Rarefaction curves showing species richness of different groups of decomposers in relation to the number of carrion-baited traps, after two days of trap presence in the forest 2024 in BEF-China. Predicted mean ± 95% CI.

**Table S2**: Taxonomic list of invertebrate decomposer species collected in carrion-baited traps in the BEF-China experiment (2023–2024, see supplementary text S2).

| **Order** | **Family** | **Genus** | **Species** | **2023** | **2024** |
| --- | --- | --- | --- | --- | --- |
| **Flies** |  |  |  |  |  |
| Diptera | Calliphoridae | Chrysomya | *Chrysomya megacephala* | 10825 | 676 |
| Diptera | Calliphoridae | Lucilia | *Lucilia porphyrina* | 7672 | 177 |
| Diptera | Calliphoridae | Hemipyrellia | *Hemipyrellia ligurriens* | 1008 | 725 |
| Diptera | Sarcophagidae | Sarcophaga | *Sarcophaga peregrina* | 619 | 1024 |
| Diptera | Calliphoridae | Chrysomya | *Chrysomya pinguis* | 1213 | 55 |
| Diptera | Muscidae | Dichaetomyia | *Dichaetomyia* sp. | 340 | 47 |
| Diptera | Calliphoridae | Lucilia | *Lucilia bazini* | 252 | 106 |
| Diptera | Muscidae | Hydrotaea | *Hydrotaea chalcogaster* | 6 | 106 |
| Diptera | Muscidae | Atherigona | *Atherigona* sp. | 40 | 11 |
| Diptera | - | - | Morphospecies #3 | 51 | 0 |
| Diptera | Muscidae | Dichaetomyia | *Dichaetomyia bibax* | 4 | 21 |
| Diptera | Muscidae | Dichaetomyia | *Dichaetomyia* sp. | 21 | 0 |
| Diptera | - | - | Morphospecies #1 | 11 | 0 |
| Diptera | - | - | Morphospecies #2 | 7 | 0 |
| Diptera | Stratiomyidae | Ptecticus | *Ptecticus aurifer* | 3 | 4 |
| Diptera | Limoniidae |  | Limoniidae | 3 | 0 |
|  |  |  |  |  |  |
| **Ants** |  |  |  |  |  |
| Hymenoptera | Formicidae | Crematogaster | *Crematogaster cf. rogenhoferi* | sampled | 1294 |
| Hymenoptera | Formicidae | Pheidole | *Pheidole nodus* | sampled | 857 |
| Hymenoptera | Formicidae | Polyrhachis | *Polyrhachis illaudata* (sensu lato) | sampled | 729 |
| Hymenoptera | Formicidae | Polyrhachis | *Polyrhachis dives* | sampled | 503 |
| Hymenoptera | Formicidae | Lepisiota | *Lepisiota* sp*.* (potentially *xichangensis*) | sampled | 375 |
| Hymenoptera | Formicidae | Camponotus | *Camponotus pseudorritans* | sampled | 342 |
| Hymenoptera | Formicidae | Pheidole | *Pheidole pieli* | - | 342 |
| Hymenoptera | Formicidae | Ectomomyrmex | *Ectomomyrmex astutus* | sampled | 211 |
| Hymenoptera | Formicidae | Aphaenogaster | *Aphaenogaster* sp. | - | 199 |
| Hymenoptera | Formicidae | Tetramorium | *Tetramorium wroughtonii* | - | 69 |
| Hymenoptera | Formicidae | Leptogenys | *Leptogenys kitteli* | - | 12 |
| Hymenoptera | Formicidae | Odonthomachus | *Odonthomachus monticola* | - | 11 |
| Hymenoptera | Formicidae | Anochetus | *Anochetus risii* | - | 2 |
|  |  |  |  |  |  |
| **Other decomposer** |  |  |  |  |  |
| Blattodae | Blattidae | Periplaneta | *Periplaneta fuliginosa* | - | 3 |
| Blattodae | Blaberidae | Pseudoglomeris | *Pseudoglomeris* sp. | - | 3 |
| Coleoptera | Staphylinidae | Aleochara | Aleochara postica | sampled | 111 |
| Coleoptera | Carabidae | Brachinus | *Brachinus sp.* | - | 1 |
| Coleoptera | Scarabaeidae | Copris | *Copris* sp. 1 | - | 8 |
| Coleoptera | Scarabaeidae | Copris | *Copris* sp. 2 | - | 93 |
| Coleoptera | Silphidae | Diamesus | *Diamesus osculans* | - | 1 |
| Coleoptera | Silphidae | Necrophila | *Necrophila cyaniventris* | sampled | - |
| Coleoptera | Silphidae | Necrophila | *Necrophila jakowlewi* | - | 1 |
| Coleoptera | Silphidae | Nicrophorus | *Nicrophorus nepalensis* | sampled | - |
| Coleoptera | Scarabaeidae | Onthophagus | *Onthophagus* sp. 1 | - | 364 |
| Coleoptera | Scarabaeidae | Onthophagus | *Onthophagus* sp. 2 | - | 48 |
| Coleoptera | Scarabaeidae | Paragymnopleurus | *Paragymnopleurus* sp. | sampled | 19 |
| Coleoptera | Curculionidae | Phytoscaphus | *Phytoscaphus* sp. | - | 4 |
| Coleoptera | Staphylinidae | Platydracus | *Platydracus brevicornis* | sampled | 2 |
| Coleoptera | Scarabaeidae | Sophrops | *Sophrops* sp. | - | 1 |
| Coleoptera | Carabidae | Trigonotoma | *Trigonotoma lewisii* | - | 1 |

**Table S2 (cont.):** Taxonomic list of invertebrate species collected in carrion-baited traps in the BEF-China experiment (2023–2024, see supplementary text S2).

| Order | Family | Genus | Species | **2023** | **2024** |
| --- | --- | --- | --- | --- | --- |
| **Other decomposer** |  |  |  |  |  |
| Dermaptera | Anisolabididae | Gonolabis | *Gonolabis* sp. | - | 14 |
| Hemiptera | Rhyparochromidae | Metochus | *Metochus* sp. | sampled | 30 |
| Lepidoptera | Geometridae | Amblychia | *Amblychia angeronaria* | - | 8 |
| Lepidoptera | Crambidae | Glyphodes | *Glyphodes* sp. | - | 34 |
| Lepidoptera | Nymphalidae | Lethe | *Lethe syrcis* | 3 | - |
| Lepidoptera | Geometridae | - | Morphospecies #4 | 45 | 46 |
| Lepidoptera | - | - | Morphospecies #5 | 9 | 17 |
| Lepidoptera | Nymphalidae | Mycalesis | *Mycalesis mineus* | 4 | 5 |
| Lepidoptera | Geometridae | Ophthalmitis | *Ophthalmitis* sp. 1 | 3 | 7 |
| Lepidoptera | Nymphalidae | Panthema | *Panthema adelma* | 18 | 4 |
| Lepidoptera | Geometridae | Parapercnia | *Parapercnia giraffata* | 1 | - |
| Lepidoptera | Geometridae | Plagodis | *Plagodis* sp. | 17 | - |
| Lepidoptera | Erebidae | Spirama | *Spirama retorta* | 7 | - |
| Lepidoptera | Erebidae | Sypnoides | *Sypnoides fumosa* | 10 | 22 |
| Orthoptera | Tettigoniidae | Atlanticus | *Atlanticus fengyangensis* | sampled | 10 |
| Orthoptera | Acrididae | Ceracris | *Ceracris kiangsu* | - | 6 |
| Orthoptera | Tettigoniidae | China | *China mantispoides* | - | 1 |
| Orthoptera | Rhaphidophoridae | Diestramima | *Diestramima excavata* | - | 33 |
| Orthoptera | Acrididae | Hieroglyphus | *Hieroglyphus annulicornis* | - | 4 |
| Orthoptera | Tettigoniidae | Mecopoda | *Mecopoda niponensis* | - | 1 |
| Orthoptera | Tettigoniidae | Meloimorpha | *Meloimorpha japonica* | - | 9 |
| Orthoptera | - | - | Morphospecies #6 | - | 1 |
| Orthoptera | Gryllidae | Xenogryllus | *Xenogryllus marmoratus* | - | 59 |
| Scutigeromorpha | Scutigeridae | - | Morphospecies #7 | - | 2 |

**Figure S6**: Relationship between the expected richness of species (i.e. species richness corrected for differences in sampling coverage) of (a-c) flies, (d-f) ants, (g-i) and other decomposers in mice carrion-baited traps after two days in the BEF-China experiment in 2024 and their abundances, tree species richness (log2-transformed) or canopy cover. Solid lines with colored intervals indicate statistically significant relationships (p < 0.05), while grey dashed lines with blurred points represent non-significant relationships. Lines show model predictions (mean), and shaded ribbons indicate 95% confidence intervals (mean ± 95% CI)

**Supplementary text 1 - total vs. conditional effects of tree species richness**

To disentangle total experimental effects from conditional relationships, we fitted two complementary models. In the BEF-China experiment, tree species richness was the primary manipulated factor. Models including tree species richness as the sole predictor quantify its total effect on the response and directly reflect the experimental design.

We also fitted models that include tree species richness alongside additional factors, such as canopy cover or decomposer diversity, which have emerged as the forest developed. These factors are linked to forest growth and are therefore partly consequences of tree species richness (Huang et al. 2018). When using Type III sums-of-squares, the effect of tree species richness is evaluated after accounting for variation explained by these covariates, which were not experimentally manipulated. As a result, these models estimate the effect of tree species richness conditional on post-treatment variables, rather than its total causal effect.

However, for carrion decomposition, microclimatic conditions associated with canopy cover are expected to be more immediate drivers than tree species richness *per se* after 15 years of stand development. We therefore focus on the extended model in the main text, while presenting the model including only tree species richness in the Supplement for transparency (Table S4-5).

|  |  | **Ants** | | | |
| --- | --- | --- | --- | --- | --- |
|  |  | Number | | Species richness | |
|  | df | χ² | p | χ² | p |
| Tree species richness | 1 | 0.26 | 0.612 | 4.60 | **0.032** |
| Residual* | 91/70 |  |  |  |  |
|  |  |  |  |  |  |
| Tree species richness | 1 | 0.08 | 0.778 | 1.14 | 0.287 |
| Canopy cover | 1 | 0.55 | 0.458 | 4.40 | **0.036** |
| Abundance | 1 |  |  | 0.63 | 0.429 |
| Residual | 90/68 |  |  |  |  |
|  |  |  |  |  |  |
|  |  | **Flies** | | | |
|  |  | Number | | Species richness | |
|  | df | χ² | p | χ² | p |
| Tree species richness | 1 | 0.29 | 0.587 | 0.11 | 0.745 |
| Residual* | 91/62 |  |  |  |  |
|  |  |  |  |  |  |
| Tree species richness | 1 | 1.62 | 0.203 | < 0.01 | 0.956 |
| Canopy cover | 1 | 16.31 | **< 0.001** | 0.31 | 0.579 |
| Abundance | 1 |  |  | 12.48 | **< 0.001** |
| Residual | 90/60 |  |  |  |  |
|  |  |  |  |  |  |
|  |  | **Other decomposers** | | | |
|  |  | Number | | Species richness | |
|  | df | χ² | p | χ² | p |
| Tree species richness | 1 | 0.13 | 0.715 | 0.03 | 0.868 |
| Residual* | 91/57 |  |  |  |  |
|  |  |  |  |  |  |
| Tree species richness |  | 0.83 | 0.364 | 0.04 | 0.849 |
| Canopy cover |  | 9.27 | **0.002** | 0.19 | 0.660 |
| Abundance |  |  |  | 12.15 | **< 0.001** |
| Residual | 90/55 |  |  |  |  |

**Table S3**: Mixed model analyses of variance of the effects of tree species richness, canopy cover (and abundance) on abundance and estimated species richness (following Chao et al. 2020) for ants, flies and other decomposers in carrion-baited traps in BEF-China in July 2025.

*Degrees of freedom refer to the models with the number / species richness of arthropods as response variable.

**Table S4**: Mixed model analyses of variance of the effects of tree species richness, canopy cover, decomposer species richness and primary decomposer (ants, flies, ants and flies, unknown) on the lost mass of mouse carcasses after two days in BEF-China in July 2025.

|  |  | % mass loss | |
| --- | --- | --- | --- |
|  |  | χ² | p |
| Tree species richness | 1 | 4.01 | **0.045** |
| Residual | 632 |  |  |
|  |  |  |  |
| Tree species richness | 1 | 1.25 | 0.264 |
| Canopy cover | 1 | 5.99 | **0.014** |
| Decomposer sp. richness | 1 | 0.82 | 0.365 |
| Primary decomposers | 3 | 57.38 | **< 0.001** |
| Residual | 627 |  |  |

**Table S5**: PERMANOVA summary on the relationship between arthropod decomposer and tree species richness, canopy cover and slope steepness in the BEF-China experiment.

| **Community (Year)** | **Predictor** | **Df** | **SS** | **R²** | **F** | **p** |
| --- | --- | --- | --- | --- | --- | --- |
| Flies in 2024 | Tree species richness | 1 | 0.20 | 0.016 | 1.32 | 0.393 |
|  | Canopy cover | 1 | 0.83 | 0.065 | 5.38 | 0.053 |
|  | Geomorph* | 4 | 0.39 | 0.031 | 0.64 | - |
|  | Residual | 73 | 11.26 | 0.883 |  |  |
| Ants in 2024 | Tree species richness | 1 | 0.38 | 0.013 | 1.17 | 0.447 |
|  | Canopy cover | 1 | 1.16 | 0.041 | 3.62 | 0.102 |
|  | Geomorph* | 4 | 2.00 | 0.070 | 1.56 | - |
|  | Residual | 77 | 13.46 | 0.902 |  |  |
| Other decomposers in 2024 | Tree species richness | 1 | 0.08 | 0.005 | 0.37 | 0.961 |
|  | Canopy cover | 1 | 1.06 | 0.071 | 5.04 | 0.070 |
|  | Geomorph* | 4 | 1.34 | 0.089 | 1.58 | - |
|  | Residual | 59 | 1.12 | 0.903 |  |  |

*The geomorphological type of the plot was included as a (‘random’) factor, in which we were not interested. Therefore, we do not present p-values.

| Order | Predictor | Chisq | Df | *P* |
| --- | --- | --- | --- | --- |
| Lepidoptera | sc.cc | 6,221642 | 1 | **0.013** |
| Lepidoptera | sc.tsr | 0,008823 | 1 | 0.925 |
| Orthoptera | sc.cc | 0,901778 | 1 | 0.342 |
| Orthoptera | sc.tsr | 3,545927 | 1 | 0.060 |
| Coleoptera | sc.cc | 17,90331 | 1 | **< 0.001** |
| Coleoptera | sc.tsr | 0,443986 | 1 | 0.505 |
| Other | sc.cc | 2,086257 | 1 | 0.149 |
| Other | sc.tsr | 0,574169 | 1 | 0.449 |

**Table S6**: Type III Wald chi-square tests of the effects of tree species richness and canopy cover on the abundance of Coleoptera (mixed models with a negative binomial model) and the presence of Lepidoptera, Orthoptera and remaining other decomposers.

**Table S7: Complete results of the piecewise structural equation model.** All specified directed paths and correlated errors are reported. Unstandardized coefficients are on each component model’s fitted scale, including the logit scale for binomial and beta models. Standardized coefficients are those returned by piecewiseSEM; blank cells indicate unavailable or non-applicable values. Correlated-error estimates represent residual associations. Marginal R² describes variance explained by fixed effects and is shown once per response. SE, standard error.
